# The Blood Flow Shutdown Induced by Combretastatin A4 Impairs Gemcitabine Delivery in a Mouse Hepatocarcinoma

**DOI:** 10.3389/fphar.2016.00506

**Published:** 2016-12-23

**Authors:** Anne-Catherine Fruytier, Cecile S. Le Duff, Chrystelle Po, Julie Magat, Caroline Bouzin, Marie-Aline Neveu, Olivier Feron, Benedicte F. Jordan, Bernard Gallez

**Affiliations:** ^1^Biomedical Magnetic Resonance Research Group, Louvain Drug Research Institute, Université Catholique de LouvainBrussels, Belgium; ^2^Institute of Condensed Matter and Nanosciences, Université Catholique de LouvainLouvain-la-Neuve, Belgium; ^3^Institut de Recherche Expérimentale et Clinique, Pole of Pharmacology, Angiogenesis and Cancer Research Laboratory, Université Catholique de LouvainBrussels, Belgium

**Keywords:** ^19^F NMR, tumor perfusion, DCE-MRI, vascular disrupting agent, drug delivery

## Abstract

In recent clinical studies, vascular disrupting agents (VDAs) are mainly used in combination with chemotherapy. However, an often overlooked concern in treatment combination is the VDA-induced impairment of chemotherapy distribution in the tumor. The work presented here investigated the impact of blood flow shutdown induced by Combretastatin A4 (CA4) on gemcitabine uptake into mouse hepatocarcinoma. At 2 h after CA4 treatment, using DCE-MRI, a significant decrease in the perfusion-relevant parameters K^trans^ and Vp were observed in treated group compared with the control group. The blood flow shutdown was indeed confirmed by a histology study. In a third experiment, the total gemcitabine uptake was found to be significantly lower in treated tumors, as assessed in a separate experiment using *ex vivo* fluorine nuclear magnetic resonance spectroscopy. The amount of active metabolite gemcitabine triphosphate was also lower in treated tumors. In conclusion, the blood flow shutdown induced by VDAs can impact negatively on the delivery of small cytotoxic agents in tumors. The present study outlines the importance of monitoring the tumor vascular function when designing drug combinations.

## Introduction

Tumor vasculature is an attractive target in anticancer therapy because of its critical role in tumor growth, development, and metastasis (Folkman, 1995). Moreover, the unique characteristics of tumor vasculature compared with that of normal tissue can be selectively exploited by antivascular therapies. Unlike antiangiogenic agents, which prevent the development of new blood vessels, vascular disrupting agents (VDAs) target the established tumor vasculature causing a rapid collapse in tumor blood flow leading to secondary extensive tumor necrosis (Thorpe, 2004; Tozer et al., 2005). Several preclinical studies have shown that VDAs induce necrosis in the poorly perfused core regions but spare tumor cells in periphery (Blakey et al., 2002; Chaplin and Hill, 2002; Siemann, 2011). This “viable rim” forms the rationale for combination approaches since VDAs affect the central part of the tumor that is often resistant to conventional therapies, whereas conventional treatments are more active on highly proliferating and well oxygenated peripheral cells (Tozer et al., 2008). Consequently, VDAs are mainly used in combination with chemotherapy in current clinical studies.

However, an often overlooked concern is the possible VDA-induced impairment of drug distribution in the tumor (Cesca et al., 2013). Indeed, currently, combination strategies are often empirical whereas a careful sequence and schedule of administration of treatments are needed to avoid negative interactions and to potentiate synergic efficacy of the drugs (Wang et al., 2012; Cesca et al., 2013). In this context, early biomarkers of response are needed to assess the impact of VDA on chemotherapy delivery and to determine optimal timing of administration in early clinical trials. Dynamic contrast-enhanced magnetic resonance imaging (DCE-MRI) is the imaging method of choice to assess early microvascular changes induced by antivascular treatments (O'Connor et al., 2012). It generates non-invasive maps of hemodynamics parameters such as blood flow and/or vascular permeability. Moreover, it has been suggested that DCE-MRI with low molecular weight gadolinium-based contrast agents can be used to predict the delivery of drugs with similar size to the interstitium of solid tumors (Artemov et al., 2001).

The first aim of the present work was to investigate how a negative modulation of tumor blood flow could affect the uptake of chemotherapeutic agents into the tumor. A second objective was to appraise the potential of DCE-MRI to predict the impact of the modulation of tumor perfusion on chemotherapy delivery. To that purpose, we first monitored the early effects of Combretastatin A4 (CA4), the lead compound of VDAs, using DCE-MRI. CA4 acts as a tubulin-binding agent, leading to a destabilization of the tubulin polymers of the cytoskeleton of proliferating endothelial cells. This way, an acute increase in tumor vascular permeability is induced, which in turn triggers several changes that together, decrease blood flow (Tozer et al., 2005). The perfusion parameters K^trans^ (the volume transfer constant between blood plasma and extravascular extracellular space) and Vp (the blood plasma volume fraction) were determined since it has been previously shown to be relevant markers of CA4 efficacy (Maxwell et al., 2002; Nielsen et al., 2010). To corroborate MRI findings, a qualitative histology study was conducted in a separate group of animals to assess the perfusion using the Hoescht 33342 marker. In parallel, the uptake of the fluorinated chemotherapeutic agent gemcitabine was assessed *ex vivo* using fluorine nuclear magnetic resonance spectroscopy (^19^F NMR).

## Materials and methods

### Animals and tumor model

Transplantable liver tumors (TLT hepatocarcinoma Taper et al., 1966) were induced i.m. into the right gastrocnemius muscle of 5-week-old male NMRI mice (Janvier, France). Tumors were allowed to reach up to 8 ± 0.5 mm in diameter prior to experimentation. For all experiments, mice were anesthetized using isoflurane (3% for induction, 1.5% for maintenance, mixed with air). Body temperature was maintained at 37.0 ± 1.0°C with a circulating water blanket and monitored together with respiration rate during experiments. All animal experiments were performed in accordance to national animal care regulations with the approval of local Ethics Board 2010/UCL/MD/01. CA4 (Sigma-Aldrich, Belgium) dissolved in DMSO was delivered i.p. at a dose of 100 mg/kg (Grosios et al., 1999).

^19^F NMR and DCE-MRI experiments were performed on separate cohorts of mice, because of the possible influence of the contrast agent on fluorine relaxation times (Ratner et al., 1989).

### DCE-MRI

Mice were divided into an untreated control group receiving vehicle (DMSO) (*n* = 6) and another treated group receiving CA4 (*n* = 6). DCE-MRI acquisition was carried out 2 h after treatment, a timing for which we anticipated an important reduction in tumor perfusion (Thorpe, 2004). The contrast agent (CtAg) used was gadoterate meglumine, a small gadolinium chelate routinely used in clinics (0.286 mmol Gd/kg). A 24G catheter was inserted in the caudal vein of mice for CtAg injection.

#### Acquisition

A quadrature whole body coil was used for radiofrequency transmission and reception. High-resolution multi-slice T2-weighted spin echo anatomical imaging was performed just before DCE-MRI. For DCE-MRI, T1 weighted gradient echo images were obtained with a fast low angle shot sequence with the following parameters: repetition time = 15 ms, echo time = 2.074 ms, flip angle = 40°, matrix = 128 × 64, field of view = 40 × 40 mm, zero-fill acceleration factor = 1.4. A first set of 400 scans with a temporal resolution of 1.19 s was acquired, with CtAg manually administered intravenously after the twentieth scan over 2 s. Afterwards, a slower DCE data set was acquired with a temporal resolution of 10.1 s to monitor the CA washout (300 images). A proton density weighted image was acquired before T1-weighted sequences with the following parameters: repetition time = 3500 ms, echo time = 2.074 ms, flip angle = 40°, matrix = 128 × 64, field of view = 40 × 40 mm.

#### Data analysis

DCE-MRI data were analyzed using the extended Tofts model (ETM). A population-averaged arterial input function was used, previously obtained in iliac artery/vein of the same mouse model (Fruytier et al., 2014a). A global region of interest (ROI) was manually delineated to cover the entire tumor area (using the T2-weighted anatomical images as reference).

The signal intensity obtained from the FLASH sequence is (Buckley and Parker, 2005):

(1) $$S=S_{0}\frac{\sin\alpha \;\cdot \;\left(1-\exp^{\frac{-TR}{T_{1}}}\right)}{\left(1-\cos\alpha \;\cdot \;\exp^{\frac{-TR}{T_{1}}}\right)}\;\cdot \;\exp\left(-\frac{TE}{T_{2}*}\right),$$

where *S*_0_ is a scaling factor comprising proton density and instrumental factors and is determined by the proton density sequence, α is the flip angle, *TR* is the repetition time, and *TE* is the echo time. Signal dependence on *T*_2_* was neglected due to the short echo time (*TE* = 2.074 ms). In tumors, the relationship between relaxation rate (1/*T*_1_) and CA concentration can be predicted by Buckley and Parker (2005):

(2) $$\frac{1}{T_{1}}=\frac{1}{T_{10}}+r_{1}\;\cdot \;C\left(t\right),$$

where *r*_1_ is the longitudinal relaxivity of the CA at 11.7T, *T*_10_ is the longitudinal relaxation time in the absence of the CA, and *C*(*t*) is the concentration of CA in tissue.

The measured tumor concentration time course [*C*_*t*_(*t*)] of pixels was individually fitted to the ETM using a Levenberg-Marquardt nonlinear least-squares procedure:

(3) $$\begin{array}{l} C_{t}(t)=K^{trans}\;\cdot \;D\;\cdot \;\sum_{i\;=\;1}^{2}a_{i}\frac{\text{exp}(-k_{ep}t)-\text{exp}(-m_{i}t)}{m_{i}-k_{ep}}\\ \;+\;v_{p}\;\cdot \;D\;\cdot \;\sum_{i\;=\;1}^{2}a_{i}\;\cdot \;\exp(-m_{i}t), \end{array}$$

where K^trans^ is the volume transfer constant between blood plasma and extravascular extracellular space (EES) [min^−1^], *v*_*p*_ is the blood plasma volume per unit volume of tissue, and *k*_*ep*_ is the rate constant between EES and blood plasma [min^−1^] (Tofts et al., 1999). D is the CA bolus dose. The constants *a*_*i*_ and *m*_*i*_ are population-averaged mean amplitudes and decay rates obtained previously in same tumor model: *a*_1_ = 16.41; *m*_1_ = 11.20; *a*_2_ = 8.41; *m*_2_ = 0.27 (Fruytier et al., 2014a). Some pixels provide nonphysiological values of *K*^trans^ (*K*^trans^ < 0, *K*^trans^ > 1 min^−1^) and/or *v*_*p*_ (*v*_*p*_ < 0, *v*_*p*_ > 1). For mean calculations, these pixels were set to zero or 1 respectively.

### *Ex vivo* ^19^F NMR

All mice received gemcitabine (800 mg/kg, IP injection, Hospira) 2 h after vehicle (*n* = 6) or CA4 treatment (*n* = 6). Tumors were carefully excised 2 h after gemcitabine treatment and snap-frozen for *ex vivo* ^19^F NMR experiments.

#### Tissue extraction

The following protocol was adapted from Refs (Olive et al., 2009; Bapiro et al., 2011). The snap-frozen tumors were weighted and homogenized in four volumes of ice-cold acetonitrile for 5 min. An equal volume of ice-cold water was added and homogenization was carried on for a further 5 min. Samples were incubated on ice for 10 min before being centrifuged at 14,000 g for 10 min at 4°C. The supernatants were transferred to vials and stored at −80°C before lyophilization.

#### Acquisition

The Freeze-dried tumor supernatants were re-suspended in 500 μl of deuterium oxide (D_2_0), spiked with 185 nmoles of 2-Fluoro-2′-deoxyadenosine (2F2dA), used as the external standard.

^19^F NMR measurements were carried out on a Bruker Avance II NMR spectrometer operating at 300 MHz for ^1^H and fitted with a 5 mm BBFO probe. 1D ^19^F spectra were acquired using inverse gated ^1^H decoupling with the following parameters: sweep width of 201 ppm, 3500 scans, acquisition time of 0.3 s, and a delay between scans of 4 s, resulting in a total measurement time of 4 h and 10 min. NMR tubes were spun at 20 Hz in the spectrometer to ensure sample homogeneity and short 1D ^1^H spectra were acquired prior to ^19^F measurements to ensure decent field homogeneity was achieved. Gain settings were kept the same for all measurements. Chemical shifts were ascertained for all components by spiking an untreated homogenate with the appropriate standards. Chemical shift referencing for ^19^F was CCl_3_F as stated in the IUPAC recommendations (Harris et al., 2002). Processing parameters prior to Fourier transformation included a 15 Hz line broadening and a backwards linear prediction in order to eliminate the broad signal due to the ^19^F background from the probe (40 points were back-predicted).

#### Data analysis

We integrated all observed fluorine peaks of gemcitabine and metabolites, using the Bruker TOPSPIN software. Two integrals were measured: one for the reference 2F2dA and the other encompassing gemcitabine and its metabolites. We normalized the integrals to the tissue wet weights in order to obtain total gemcitabine metabolite concentration in samples in μg/mg tissue.

### Histology

Six NMRI tumor-bearing mice were treated with vehicle (*n* = 3) or CA4 (*n* = 3). Two hours after treatment, the functional perfusion marker Hoechst 33342 (15 mg/kg; iv injection; Sigma-Aldrich) was injected. Mice were sacrificed 2 min later. Five micrometer tumor cryosections were immunostained with a rat monoclonal antibody against CD31 (BD, clone MEC 13.3) and revealed with Alexa568 conjugated anti-rat secondary antibodies (Invitrogen). Tumor sections were imaged using a Zeiss Axioimager Z1 fluorescent microscope equipped with an Apotome module (Zeiss, Wetzlar, Germany).

Estimation of CA4-induced cell death was also obtained from TLT tumor-bearing mice treated with CA4 (*n* = 7) or vehicle (*n* = 8). Tumors were excised at day 1 after treatment initiation. Tumors were embedded in Tissue-Tek OCT compound and frozen in liquid nitrogen-cooled isopentane for cryosectioning. Samples were cut into 5 μm sections. The frozen slices were stained with Haematoxylin & Eosin or were probed for cellular death by TUNEL assay using an in situ cell death detection kit (Roche Diagnostics, Belgium). For TUNEL assay, nuclei were also counterstained with Hoechst 33342. Slides were then scanned with a Zeiss Mirax fluorescence microscope. Cell death was quantified using Frida software and expressed as a percentage of the whole tumor area.

### Statistical analysis

Values are presented as mean ± standard error, unless otherwise stated. All statistical analysis was performed with GraphPad Prism 5. Non parametric Mann-Whitney tests were used to compare mean changes between control and treated groups. *P* < 0.05 are considered statistically significant.

## Results

### CA4 treatment induced an early decrease in tumor blood flow in TLT tumors

**Figure 1:**
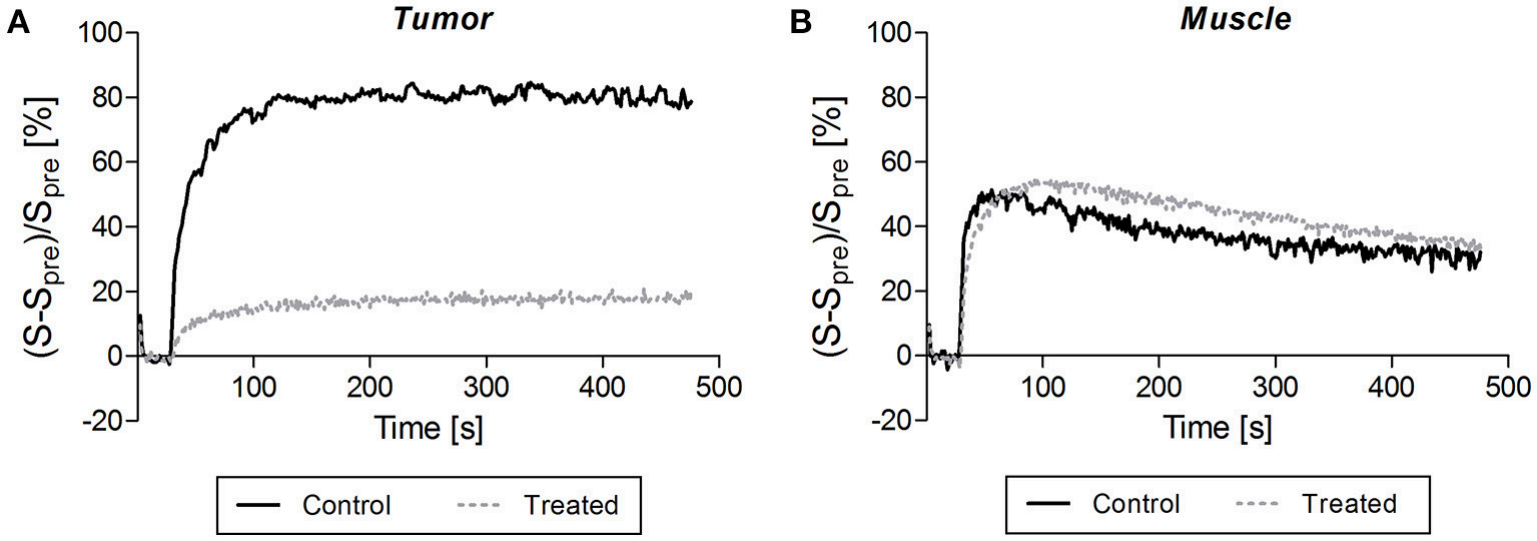
**Typical signal enhancement following contrast agent injection observed in tumor (A)** and in muscle **(B)** of a control mouse (dark solid line) and a treated mouse (gray dotted line). **(A)** A smaller enhancement and a slower washout were typically observed in treated compared to untreated tumors. **(B)** By contrast, the enhancement pattern was similar in muscle of control and treated mice.

**Figure 2:**
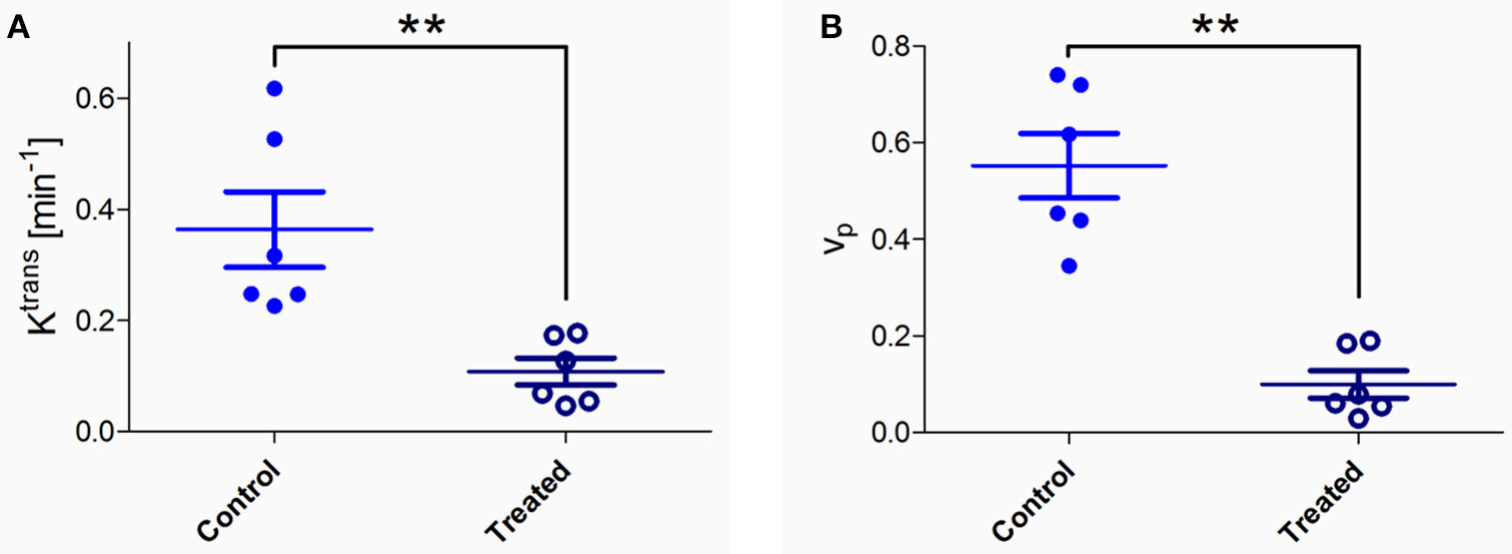
**(A)** Absolute values of pharmacokinetic parameters K^trans^ for control and treated tumors. A significant K^trans^ decrease was observed in treated tumors compared to untreated ones (Mann-Whitney test; ^**^p < 0.01). **(B)** Values of Vp (plasma volume fraction) for control and treated tumors. A significant decrease in Vp was observed in treated tumors compared to untreated ones (Mann-Whitney test; ^**^p < 0.01).

Figures 1, 2 illustrate DCE-MRI findings. DCE-MRI consists of the acquisition of T1-weighted images before, during, and after a contrast agent administration allowing for the generation of a signal intensity-time course from a tissue of interest. Figure 1 shows examples of enhancement curves (percentage of signal enhancement vs. time of selected region of interest) obtained from tumor (Figure 1A) and muscle (Figure 1B) of a control and a treated mouse. A smaller enhancement and a slower washout were typically observed in treated compared to untreated tumors (Figure 1A). By contrast, the enhancement pattern was similar in muscle of control and treated mice (Figure 1B).

The pharmacokinetic modeling of pixel signal intensity-time curve allowed extracting K^trans^ and Vp for each pixel. Mean tumor K^trans^ and Vp were calculated for each mouse by averaging all pixel values (Figure 2). A significant K^trans^ decrease was observed in treated tumors compared to untreated tumors (0.11 vs. 0.36 min^−1^; ^**^p < 0.01; Figure 2A). K^trans^ values were lower in treated mice, particularly in tumor core regions. A significant Vp decrease was also observed in treated tumors compared to untreated tumors (0.099 vs. 0.552; ^**^p < 0.01) (Figure 2B).

**Figure 3:**
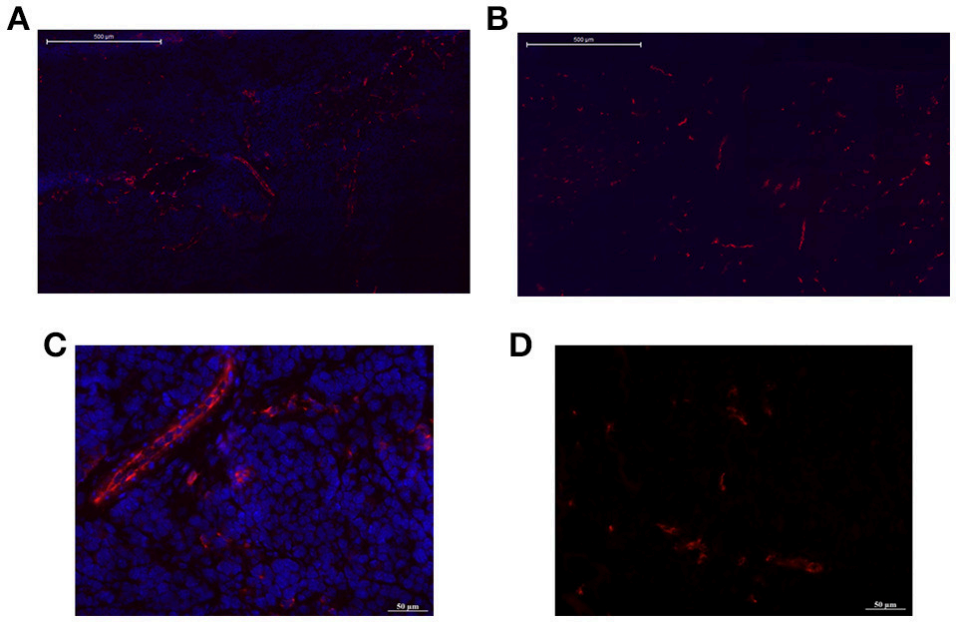
**Representative pictures of tumor sections from untreated and CA4-treated mice stained with CD31 antibody for endothelial cells (red)**. High (bar = 500 μm) and low (bar = 50 μm) magnifications are presented. The Hoechst 33342 perfusion marker was administered 2 min before sacrifice. Treated tumors **(B,D)** showed less perfused vessels (blue) compared to untreated tumors **(A,C)**.

**Figure 4:**
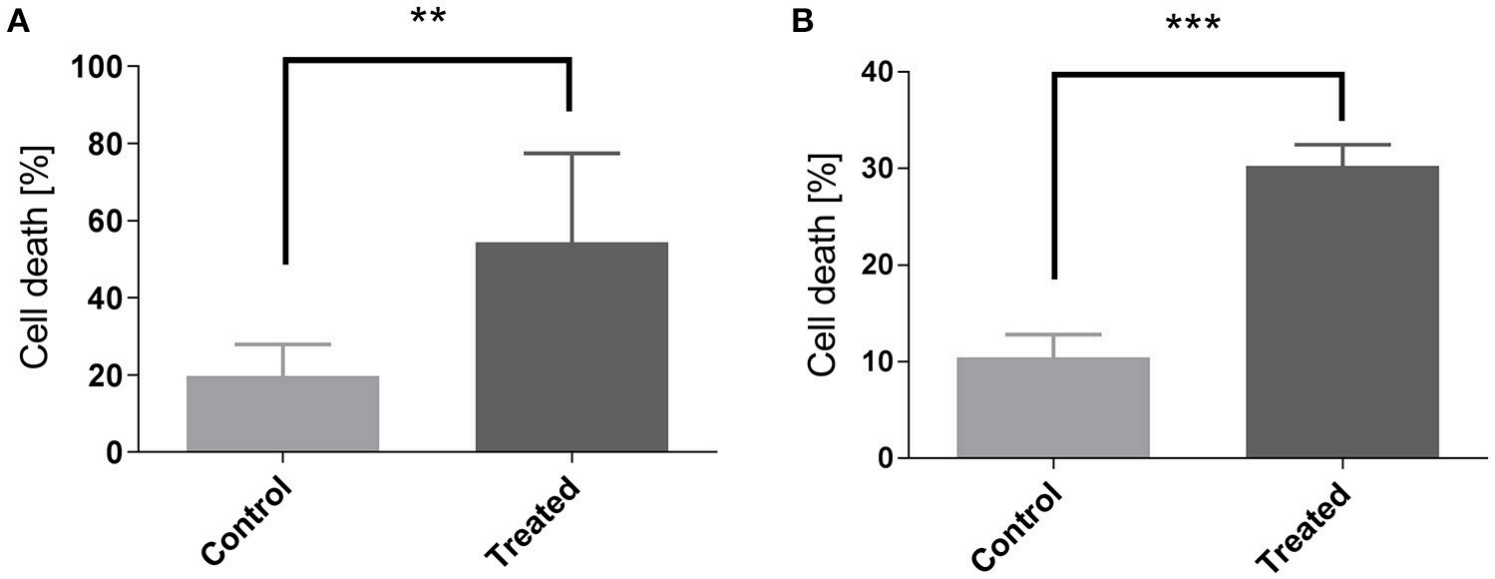
**Measurement of cellular death by quantification of Hematoxylin Eosine staining (A)** and of TUNEL assay **(B)** in control and in CA4-treated tumors at day 1. Data are expressed as means ± SEM (Unpaired *t*-tests; ^**^p < 0.01, ^***^p < 0.001).

Histology confirmed the blood flow shutdown seen by DCE-MRI induced by CA4 treatment (Figure 3). Treated tumors (Figures 3A,C) showed less perfused vessels (assessed with Hoechst 33342, administered 2 min before sacrifice) compared to untreated tumors (Figures 3B,D). No difference was visually observed between groups in vascular density (assessed with CD31 antibody). We also observed an increase in tumor cell death 1 day after CA4 treatment. Histological analysis of Haematoxylin/Eosin -stained slices showed that tumors treated with CA4 present enlarged necrotic regions (54 ± 9%) as compared with tumors treated with vehicle (20 ± 3%) (^**^p < 0.01) (Figure 4A). On the other hand, using the TUNEL assay (Figure 4B), positively stained areas were increased in treated tumors compared to control tumors (10 vs. 30%; ^***^p < 0.001; Figure 4B).

### CA4 treatment induced a decrease of gemcitabine uptake in TLT tumors

**Figure 5:**
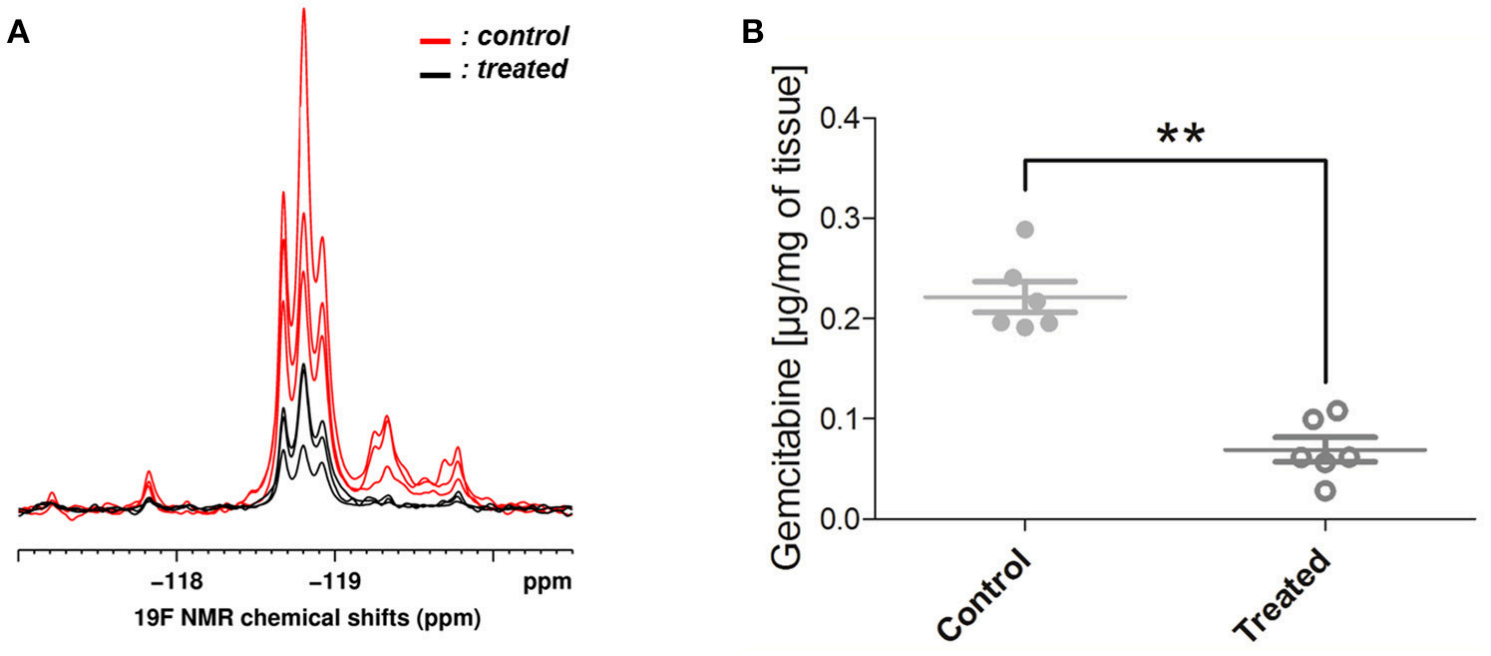
**^19^F NMR findings. (A)** Typical spectra obtained from 3 treated (in black) and 3 untreated (in red) tumor homogenates. Gemcitabine (dFdC) and two of its metabolites, 2′,2′-difluorodeoxyuridine (dFdU) and gemcitabine triphosphate (dFdCTP), were identified on the spectra. The metabolite dFdCTP was absent (or present in smaller quantities) in treated tumors. **(B)** Total gemcitabine uptake was quantified using an integral encompassing gemcitabine and its metabolites. A significant decrease of total gemcitabine uptake was observed in treated tumors when compared with untreated ones (Mann-Whitney test; ^**^p < 0.01).

**Figure 6:**
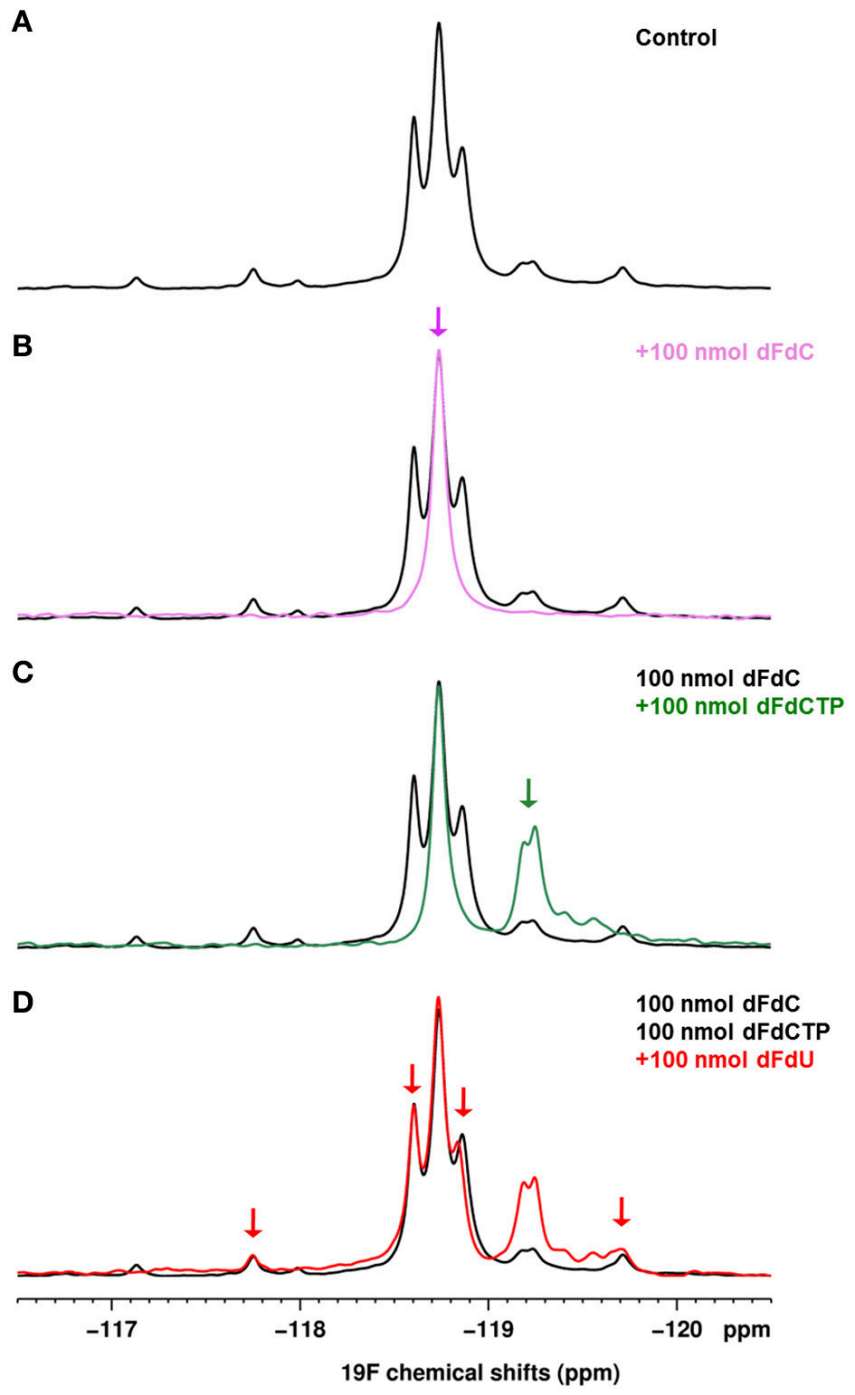
**(A)** 1D ^19^F spectrum of a control tumor homogenate (800 mg/kg gemcitabine). **(B–D)** 1D ^19^F spectra of an untreated homogenate spiked successively with 100 nmoles of dFdC **(B)**, dFdCTP **(C)**, dFdU **(D)**. Each spectrum is overlaid on the spectrum of the control tumor homogenate shown in **(A)**.

In *ex vivo* ^19^F NMR experiments, gemcitabine parent compound (dFdC) and two of its metabolites, 2′,2′-difluorodeoxyuridine (dFdU) and gemcitabine triphosphate (2,2′-difluorodeoxycytidine-5′-triphosphate-dFdCTP) were identified. Typical spectra are shown on Figure 5A. We quantified the total gemcitabine amount using an integral encompassing gemcitabine and its metabolites. A significant decrease of total gemcitabine amount was observed in treated tumors when compared with untreated ones (0.07 vs. 0.22 μg/mg of tissue; ^**^p < 0.01; Figure 5B). Chemical shifts were determined by spiking an untreated tumor homogenate with appropriate standards. Results are shown on Figure 6. Respective assignments in ^19^F NMR spectra were −54.6 ppm for external standard 2F2dA, −118.7 ppm for dFdC, two doublets at −118.2 and −119.25 ppm (*J* = 241 Hz; i.e., four frequency lines at −117.8, −118.6, −118.8, and −119.7 ppm) for dFdU, and a doublet at −119.25 ppm (*J* = 15 Hz) for dFdCTP. One can observe that the metabolite dFdCTP was present in smaller quantities in treated tumors compared to untreated tumors (Figure 5). For quality assurance purpose, we measured the area under the curve of the external standard 2F2dA for each sample and observed that it was stable from experiment to experiment, with a standard deviation of <5% of the mean.

## Discussion

In this work, we demonstrate that a negative modulation of tumor blood flow by CA4 treatment causes a significant decrease in gemcitabine delivery in a solid tumor mouse model. It outlines the need to assess the impact of treatments on the tumor vascular function, for instance using DCE-MRI, when appraising a novel drug combination.

CA4 acts as a tubulin-binding agent, leading to a destabilization of the tubulin polymers of the cytoskeleton of proliferating endothelial cells. This way, an acute increase in tumor vascular permeability is induced, which in turn triggers several changes that together, decrease blood flow (Tozer et al., 2005). DCE-MRI confirmed the blood flow shutdown at 2 h after CA4 treatment, as revealed by reduced enhancement patterns as well as K^trans^ and Vp decrease in treated tumors, in comparison to untreated ones (Figure 2). The parameter K^trans^ represents the rate of transfer of the CtAg from the blood to the interstitial space. The physiological interpretation depends on the balance between capillary permeability and blood flow in the tumor: higher permeability makes K^trans^ more reflective of blood flow (flow-limited situation), whereas lower permeability makes K^trans^ more reflective of vascular permeability (permeability-limited situation; Tofts et al., 1999; O'Connor et al., 2007; Zweifel and Padhani, 2010). However, we previously reported that, in case of VDA assessment, K^trans^ changes determined with small molecular weight CtAg are more reflective of tumor blood flow changes, due to the high permeability of the CtAg across the capillary membrane (corresponding to a flow-limited situation; Fruytier et al., 2014b). Here, the observed K^trans^ decrease (Figure 2A) can thus be related to the early tumor blood flow shutdown induced by CA4, as confirmed by histology. The observed decrease in Vp (Figure 2B), the plasma volume fraction, is also consistent with the tumor blood flow shutdown. Of note, histological studies confirmed that CA4-treated tumors were less perfused compared to untreated tumors (Figure 3). As a consequence of this blood flow shutdown, we also observed that tumors treated with CA4 present more necrotic and apoptotic areas 24 h post-treatment (Figure 4).

In a second step, we investigated how the induced tumor blood flow shutdown can impact the delivery of chemotherapy, using *ex vivo* ^19^F NMR spectroscopy to determine the levels of gemcitabine and its major metabolites in tumors. Gemcitabine is a prodrug that must be phosphorylated intracellularly by deoxycytidine kinase to exhibit cytotoxic activities. Its major intracellular active metabolite is dFdCTP, although it remains in a constant ratio with gemcitabine monophosphate and diphosphate (Heinemann et al., 1992). Gemcitabine is inactivated mainly by deoxycytidine deaminase mediated conversion to dFdU (Mini et al., 2006). The ^19^F NMR experiments showed that the total gemcitabine uptake into the tumor was significantly lower in treated mice (CA4+gemcitabine) compared to controls (vehicle+gemcitabine) (Figure 5). These results are in line with those of Kristjansen et al. which demonstrated in *ex vivo* perfused human small cell lung cancer that the initial intratumor distribution of dFdC is flow dependent (Kristjansen et al., 1996). Gemcitabine, its three phosphorylated metabolites and dFdU overlap in ^19^F spectrum, making accurate quantification for individual metabolites difficult (Blackstock et al., 2001). However, it is clear that the intensity of the peak of the most active metabolite dFdCTP was lower in treated tumors compared to controls, or in some cases, null.

Several studies reported that blood flow reductions following VDA treatment could potentiate chemotherapy efficacy by trapping chemotherapy within the tumor and consequently increasing tumor exposure to the drug (Siim et al., 2003; Siemann, 2011). This has been observed for combinations where VDA was given after chemotherapy but also for VDA given before chemotherapy. For instance, Pruijn et al reported that the administration of the antivascular agent 5,6-dimethylxanthenone-4-acetic acid 2 h before the chemotherapeutic agent melphalan leads to an increase of the alkylating agent levels in the tumor and consequently to an enhanced activity (Pruijn et al., 1997). Additional studies reported that concurrent administration of VDA and chemotherapy increased antitumor activity (Morinaga et al., 2003; Kleespies et al., 2005).

However, as outlined in the present work, the induced blood flow shutdown could also decrease drastically the delivery of the chemotherapeutic agent inside the tumor. The potentiation of chemotherapy by drug trapping will take place only if the chemotherapeutic agent can reach the tumor before the vascular shutdown. Predictive biomarkers of drug delivery could thus be particularly useful in assessing novel drug combinations. For that purpose, we appraised the potential of DCE-MRI using low molecular weight CtAg which could mimic small conventional cytotoxic drugs in such a way that their delivery inside the tumor could be reflective of uptake and distribution of conventional chemotherapy (Artemov et al., 2001). In addition to be a pharmacodynamic biomarker of CA4 treatment, the DCE-MRI parameter K^trans^ was predictive of the gemcitabine delivery. We observed a similar decrease of K^trans^ and gemcitabine delivery in the parallel groups of mice (70 and 68.7% of decrease, respectively).

Response to chemotherapy is determined by the delivery of cytotoxic agent to the tumor but also by chemotherapy cellular uptake, retention, and metabolic activation, as well as by chemotherapy sensitivity in the target tumor cells (van Laarhoven et al., 2007; Jain, 2012). Moreover, in addition to any spatial cooperation between both treatments, final outcome following treatment combination will be also affected by the effect of the VDA on the tumor (cell/vasculature; Wang et al., 2012). The microenvironmental changes secondary to VDA-induced blood flow shutdown can also modulate the sensitivity of chemotherapy (Pruijn et al., 1997; Siim et al., 2003). Indeed, on the one hand, hypoxia and acidosis could be favorable to the activity of specific agents (e.g., bioreductive drugs, melphalan; Pruijn et al., 1997; Taraboletti et al., 2010), whereas, the severe glucose and oxygen deprivation induced by CA4P have been shown to induce GRP78 expression that could lead to drug resistance (Dong et al., 2005).

Our study could be considered as a proof-of-concept that directly links the effect of VDA on the blood flow to the delivery another chemotherapeutic agent. A limitation of our study is that we analyzed this effect only at one timing (2 h) post-VDA treatment. For the future, it could be interesting to follow the dynamics of this effect at different timings and to study the impact of the combination at different timings on the tumor response to treatments.

In conclusion, although the link between drug delivery and therapeutic efficiency is not straightforward, the present study demonstrates that the potential impact of VDA on intratumoral drug delivery should be taken into account when designing novel drug combination. For that purpose, the assessment of tumor vascular function by DCE-MRI could be helpful to avoid misinterpretation when a failure of treatment is observed, but also to maximize therapeutic gain.

## Author contributions

AF conceived and designed the work, acquired, analyzed and interpreted data, drafted and revised the manuscript, approved the final version. CL, CP, JM, CB, and MN acquired, analyzed and interpreted data, revised the manuscript, approved the final version. OF and BJ analyzed and interpreted data, revised the manuscript, approved the final version. BG conceived and designed the work, analyzed and interpreted data, drafted and revised the manuscript, approved the final version. All authors agree to be accountable for all aspects of the work.

### Conflict of interest statement

The authors declare that the research was conducted in the absence of any commercial or financial relationships that could be construed as a potential conflict of interest.
